# Association of sociodemographic and oncological features with decision on implant‐based versus autologous immediate postmastectomy breast reconstruction in Chinese patients

**DOI:** 10.1002/cam4.2133

**Published:** 2019-04-05

**Authors:** Zhuming Yin, Yan Wang, Jingyan Sun, Qingfeng Huang, Jing Liu, Shanshan He, Chunyong Han, Shu Wang, Bowen Ding, Jian Yin

**Affiliations:** ^1^ Department of Breast Oncoplastic Surgery Tianjin Medical University Cancer Institute and Hospital Tianjin China; ^2^ National Clinical Research Center for Cancer Tianjin China; ^3^ Key Laboratory of Breast Cancer Prevention and Therapy Tianjin Medical University, Ministry of Education Tianjin China; ^4^ Key Laboratory of Cancer Prevention and Therapy Tianjin China; ^5^ Tianjin’s Clinical Research Center for Cancer Tianjin China; ^6^ Sino‐Russian Joint Research Center for Oncoplastic Breast Surgery Tianjin China

**Keywords:** breast cancer, decision‐making, immediate breast reconstruction, oncological features, sociodemographic characteristics

## Abstract

**Background and objectives:**

Immediate postmastectomy breast reconstruction (IPBR) has gained wide popularity in China. We sought to clarify the prevalence and predictors of implant‐based vs autologous IPBR among Chinese patients.

**Methods:**

A retrospective cohort study was performed using a prospectively maintained database. Women who underwent IPBR during 2001‐2017 were included. The modality‐specific trends were deciphered by curve fitting analysis. The association of sociodemographic and oncological features with the decision for implant‐based vs autologous IPBR was investigated using multivariate logistic regression and structural equation modeling.

**Results:**

Among 905 patients included in the study, 479 underwent implant‐based IPBR and 426 underwent autologous procedures. The implant/autologous ratio has increased exponentially over time. Multivariate analysis demonstrated that unmarried patients with BMI ≤ 24 kg/m^2^, earlier clinical tumor stage, and preoperative pathological diagnosis of noninvasive lesion are more likely to choose implant‐based IPBR compared to autologous procedures. The indirect effects of age, mastectomy type, and neoadjuvant chemotherapy were further demonstrated by the structural equations.

**Conclusions:**

The sociodemographic and oncological features are directly or indirectly associated with the decision on type of IPBR. The findings may facilitate both patients and physicians to make a high‐quality decision by holistic evaluation of the sociodemographic and oncological features.

## 1 INTRODUCTION

With the increasing incidence of breast cancer and remarkably improved survival,^1^, ^2^ a growing number of patients in China have to contend with the long‐term effects of surgery on body image and quality of life during their lifetimes. Immediate postmastectomy breast reconstruction (IPBR) is therefore a strongly recommended treatment option for patients undergoing mastectomy for cancer not only to restore a breast mound, but more importantly to relieve the postoperative stress without affecting the prognosis or detection of locoregional recurrence.^3^

Recently, considerable attention has been given to the decision on whether to undergo IPBR, which is substantially affected by patients' clinicopathological characteristics,^4^, ^5^, ^6^ knowledge, and perceptions,^7^, ^8^ as well as the government policy.^9^, ^10^ Nevertheless, the majority of patients also linger on the reconstructive modality because the predictors associated with the reconstructive procedure choice remain speculative and empirical. Previous outcome studies have focused largely on the postoperative complications,^11^, ^12^, ^13^ the patient‐reported outcomes,^14^, ^15^, ^16^ and the health‐care expenditures,^17^ which may help new patients understand the expected results and costs, and then make informed decisions on the type of reconstruction. However, these factors cannot be determined before surgery when the decision must be made. Therefore, the preoperative information, such as sociodemographic characteristics and oncological features, should be taken into account as the main reference for the reconstructive procedure choice. Unfortunately, few studies have systematically elicited these information specific to the decision on the type of reconstruction, especially in Asian patients.

Hence in the current study, we sought to investigate the sociodemographic characteristics and oncological features with respect to the decision on implant‐ vs autologous‐based IPBR among Chinese patients. The results of the present study may assist both patients and physicians to make optimized decisions regarding a reconstructive modality which is most consistent with the personal traits of patients.

## 2 METHODS

### 2.1 Study design

A retrospective cohort study was performed using a prospectively maintained database, which comprised all the female patients undergoing mastectomy in the setting of the National Clinical Research Center for Cancer and the largest breast cancer center in China. Women who underwent IPBR from 1 January 2001 to 31 December 2017 were identified, and their sociodemographic characteristics, oncological features, and decisions on reconstructive paradigm were collected. The missing data were retrieved by review of the medical records. This study was approved and deemed exempt from personal informed consent requirements by the Ethics Committee of Tianjin Medical University Cancer Institute and Hospital.

### 2.2 Study population

**Figure 1:**
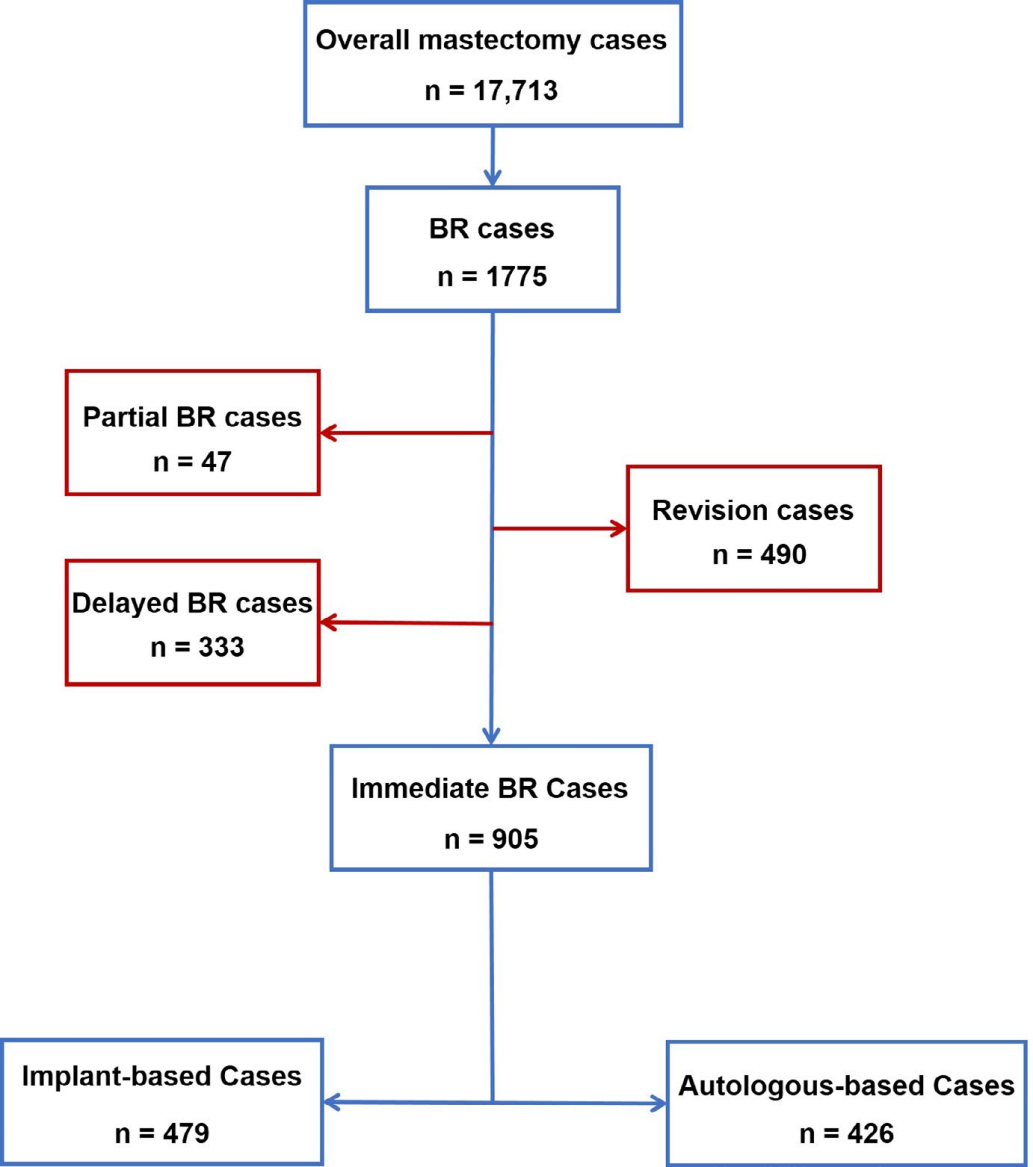
Flow chart of patient inclusion. The decay in the cohort is shown from initial enrollment of patients through the selection of the cases for the ultimate analysis in this study. Abbreviation: BR, breast reconstruction

Patients were eligible for the current study if they met all the following inclusion criteria: 18 years or older on the date of surgery, diagnosis as benign lesion or stages 0 through III breast cancer, documentation of IPBR during the study period. Cases were excluded from the study if they met at least one of the following criteria: stage IV breast cancer, neoplasms with other malignant diagnoses (eg, sarcoma), reconstruction following breast conserving surgery or partial mastectomy, delayed or tertiary breast reconstruction, nipple reconstruction, or revision surgery. The inclusion and exclusion methodology is further described in Figure 1. The patient education and decision aid were conducted by well‐trained residents and nurses after hospitalization and before surgery according to a handbook with uniform format.

### 2.3 Measures

The annual amount of implant‐ and autologous‐based IPBRs was recorded to illustrate the trend in the reconstructive paradigm shift. The sociodemographic variables included age, body mass index (BMI), marital status, and residency. Age (≤40 years vs >40 years) and BMI (≤24 kg/m^2^ vs >24 kg/m^2^) were categorized in a dichotomous manner according to the median age and the standard of overweight, respectively. Due to the absence of personal income in the database, the economic status of the patients was reflected by the economic development of their residency. The developed province‐level regions were defined with the per‐capita disposable income over 25 000 CNY in 2016, which included the regions of Shanghai, Beijing, Zhejiang, Tianjin, Jiangsu, Guangdong, Fujian, and Liaoning according to the national data.^18^ To investigate the impact of the accessibility of medical resources on the reconstructive procedure choice, the location of residency was involved, and the patients were divided into local and nonlocal groups depending on whether they lived in Tianjin city where our center locates. The oncological features included the family history of breast or ovarian cancer, history of abdominal surgery, clinical tumor staging, laterality (uni‐ vs bilateral procedures), type of mastectomy (modified radical mastectomy vs nipple‐ or skin‐sparing mastectomy), preoperative pathological diagnosis (noninvasive lesion vs invasive cancer), and neoadjuvant chemotherapy. The postmastectomy radiotherapy was not involved because it can only be determined by the postoperative pathology, even though it is usually predicted by the clinical tumor staging and preoperative pathology, both of which have already been included in the current study.

The types of IPBR were grouped into implant and autologous categories based on the *Clinical Modification of International Classification of Diseases, Ninth Revision, volume 3* (*ICD‐9‐CM‐3*, See details in the Table S1). All the patients undergoing IPBR had access to a full range of reconstructive choices during the study period, including implant‐based and autologous procedures. Patients undergoing expander/implant procedures were included in the implant group unless the autologous tissue was used instead of implant after expansion, which should be categorized as the autologous group. Cases involving both the reconstructive modalities (eg, Combination of latissimus dorsi flap transfer and implant insertion) were included in the implant group.

### 2.4 Statistical analysis

The sociodemographic and oncological features were displayed using proportions for categorical variables and means with standard deviations (SDs) or medians with interquartile ranges (IQRs) for continuous variables. The trend in the ratio of implant vs autologous procedures was evaluated by curve fitting analysis using Sigmaplot version 13 (Systat Software Inc, Chicago, IL). Differences of the variables between the implant and autologous group were assessed by Pearson's chi‐squared test because all the variables were categorized. Variables with a value of *P* < 0.05 in the Pearson's chi‐squared test were included as covariates in the multivariate logistic regression analysis to estimate the odds ratios (ORs) and 95% confidence intervals (95% CIs) for the association between sociodemographic and oncological features and decisions on the type of reconstruction. The sensitivity and specificity of the regression model were tested by receiver operating characteristic (ROC) curve analysis using MedCalc version 17.0 (MedCalc Software Inc, Ostend, Belgium).

The structural equation modeling (SEM) was employed to clarify the indirect relationship between the study variables and the reconstructive procedure choice. First, the Pearson and Spearman rank correlation analyses were performed between study variables. Then path analysis and confirmatory factor analysis were used to establish the SEMs using SPSS Amos version 21.0 (IBM Inc, Armonk, NY). The model fit was tested based on the following rules^19^: (a) χ^2^ value: a lower χ^2^ or χ^2^/*df* value and a nonsignificant *P*value (*P* > 0.05) indicate improved model fit; (b) RMSEA (the root mean square error of approximation): best if below 0.05; and (c) CFI (the comparative fit index): best if above 0.95. The mediating and moderating effect of the variables in the SEMs were assessed by the Preacher and Hayes method.^20^, ^21^ All the analyses were conducted using SPSS version 22.0 (IBM Inc, Armonk, NY) unless otherwise stated. Significance was assumed at *P* < 0.05 based on two‐sided tests.

## 3 RESULTS

During the study period, 17 713 patients received mastectomy for breast cancer or benign lesions according to the search result of the database. Of these, a total of 1 775 cases undergoing breast reconstruction were eligible for initial sample selection. Of this total, 47 (2.6%) cases underwent partial reconstruction of the breast, 333 (18.8%) cases received delayed reconstruction, and 490 (27.6%) were revision cases; all were excluded from further analyses. The remaining IPBR cases were performed by 20 surgeons respectively, and 21 expander/implant cases and 5 expander/abdominal flap cases were identified. Therefore, the final cohort consisted of 905 patients, of whom 479 underwent implant‐based procedures and 426 were included in the autologous group.

**Figure 2:**
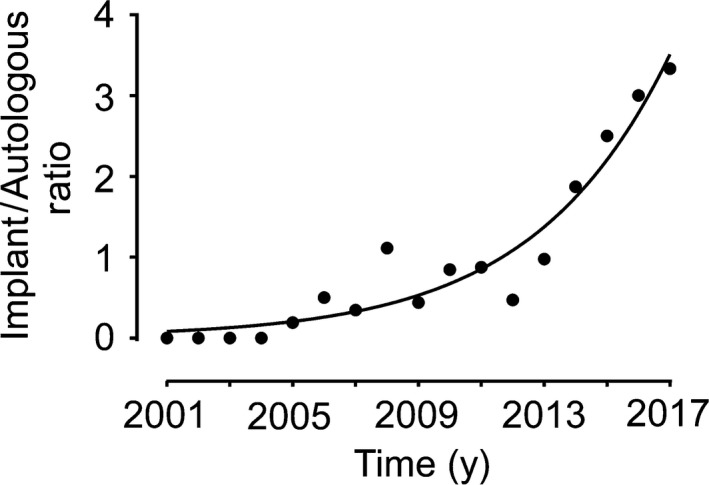
Temporal trends in the use of implant‐ vs autologous‐based immediate postmastectomy breast reconstruction. The implant/autologous ratio increased in an exponential manner (*R* ^2^ = 0.87, *P* < 0.01) among the patients in the current cohort

The median age of the final cohort was 40 (IQR, 35‐45) years, while the median BMI was 22.48 (IQR, 20.58‐24.43) kg/m^2^. The trend in the reconstructive modality changed over time (Figure 2). A preponderance of autologous tissue procedures was observed early in the study period, but the patients were more than three times as likely to decide to undergo implant‐based IPBR in 2017 (*P* for trend < 0.01). The curve fitting analysis indicated that the increase in the implant/autologous ratio better fits an exponential (*R* ^2^ = 0.87, *P* < 0.01) as opposed to a linear pattern (*R* ^2^ = 0.75, *P* < 0.01).

The main characteristics of the study cohort, stratified by type of breast reconstruction, are shown in Table 1. Significant differences were revealed concerning age, BMI, marital status, clinical tumor staging, laterality, type of mastectomy, preoperative pathological diagnosis, and neoadjuvant chemotherapy between the implant and autologous group (*P* < 0.05). On the contrary, the distinctions of economic development and location of residency, family history of breast or ovarian cancer, and history of abdominal surgery did not approach significance (*P* > 0.05). The reconstructive procedure choice varied dramatically between the episodes of 2001‐2012 and 2013‐2017 (*P* < 0.01), which is dichotomized according to the median year of surgery, indicating the need to adjust for the confounder in the following analysis.

**Table 1:** Sociodemographic and oncological features of breast cancer patients undergoing IPBR (n = 905)^a^

	Implant (n* = *479)	Autologous (n = 426)	*P* value^b^
Surgery years			<0.001
2001‐2012	154 (32.2)	274 (64.3)	
2013‐2017	325 (67.8)	152 (35.7)	
Age			0.009
≤40 years	260 (54.3)	194 (45.5)	
>40 years	219 (45.7)	232 (54.5)	
BMI			<0.001
≤24 kg/m^2^	379 (79.1)	268 (62.9)	
>24 kg/m^2^	100 (20.9)	158 (37.1)	
Marital status			<0.001
Single	68 (14.2)	17 (4.0)	
Married or coupled	411 (85.8)	409 (96.0)	
Residency (economic development)			0.727
Developed	279 (58.2)	253 (59.4)	
Undeveloped	200 (41.8)	173 (40.6)	
Residency (location)			0.101
Local	179 (31.4)	182 (42.7)	
Nonlocal	300 (62.6)	244 (57.3)	
Family history of breast or ovarian cancer			0.256
No	450 (93.9)	392 (92.0)	
Yes	29 (6.1)	34 (8.0)	
History of abdominal surgery			0.839
No	357 (74.5)	320 (75.1)	
Yes	122 (25.5)	106 (24.9)	
Clinical tumor staging^c^			<0.001
0	45 (9.4)	21 (4.9)	
I	171 (35.7)	103 (24.2)	
IIA	188 (39.2)	200 (47.0)	
IIB	60 (12.5)	69 (16.2)	
IIIA	15 (3.2)	25 (5.9)	
IIIB	0 (0)	8 (1.8)	
Laterality			<0.001
Unilateral	458 (95.6)	424 (99.5)	
Bilateral	21 (4.4)	2 (0.5)	
Type of mastectomy			0.008
Modified radical mastectomy	221 (46.1)	234 (54.9)	
Nipple‐sparing mastectomy^d^	258 (53.9)	192 (45.1)	
Preoperative pathological diagnosis			<0.001
Noninvasive lesion	143 (29.9)	70 (16.4)	
Invasive cancer	336 (70.1)	356 (83.6)	
Neoadjuvant chemotherapy			0.021
No	411 (85.8)	340 (79.8)	
Yes	68 (14.2)	86 (20.2)	

IPBR, immediate postmastectomy breast reconstruction; SD, standard deviation; BMI, body mass index.

a The data are displayed as No. (%).

b χ^2^ *test.*

c The tumor staging is determined according to the AJCC Cancer Staging Manual (7th edition).

d Skin‐sparing mastectomy is also included in this type.

**Figure 3:**
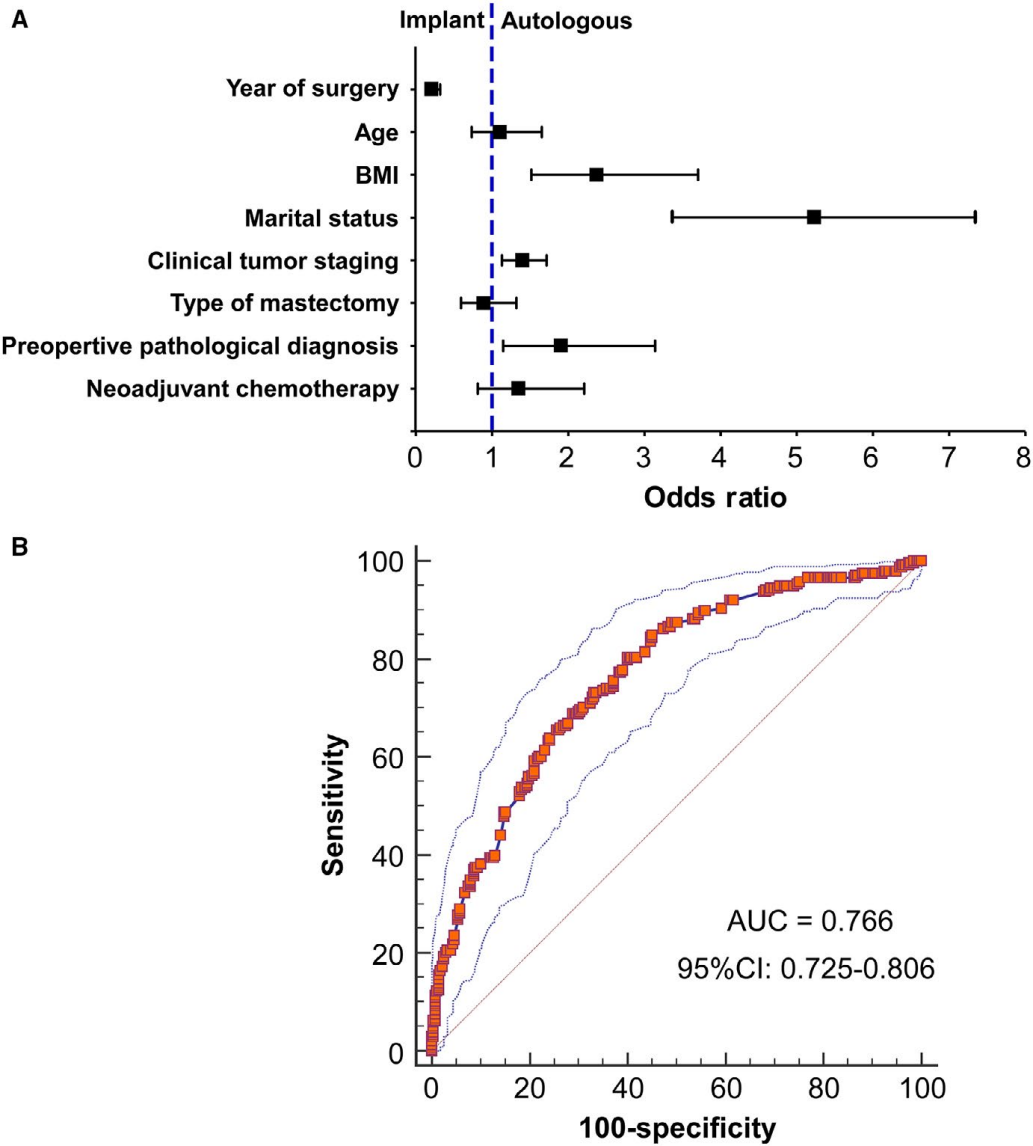
Multivariate logistic regression analysis. (A) The likelihood of having autologous‐(1) vs implant‐based (0) breast reconstruction was compared (n* = *905). BMI, marital status, clinical tumor staging, and preoperative pathological diagnosis were independently associated with the decision on type of breast reconstruction in the adjusted model. (B) The sensitivity and specificity of the regression model were determined by receiver operating characteristic (ROC) curve. The areas under curve (AUC, 0.766; 95% CI, 0.725‐0.806) suggested satisfying model fitting

The variables with significant difference (*P* < 0.05) in the univariate comparison across decisions on type of reconstruction were involved in the subsequent multivariate logistic regression modeling, adjusted for the year of surgery. Laterality was removed from the multivariate model because the sample size of bilateral cases using autologous procedures (2[0.5%]) was not sufficient to avoid selection bias. As shown in Figure 3A, BMI > 24 kg/m^2^ (adjusted OR, 2.38; 95% CI, 1.52‐3.71; *P* < 0.01), being married or coupled (adjusted OR, 5.22; 95% CI, 3.30‐7.35; *P* < 0.01), having later clinical stage (adjusted OR, 1.40; 95% CI, 1.14‐1.72; *P* < 0.01), and preoperative pathological diagnosis of invasive cancer (adjusted OR, 1.91; 95% CI, 1.16‐3.15; *P* < 0.05) were independently associated with the decision for autologous breast reconstruction. The model performed well in predicting the likelihood of having implant‐based vs autologous breast reconstruction among the patients in the current cohort (Figure 3B; areas under ROC curve, 0.766; 95% CI, 0.725‐0.806).

To figure out the indirect effects of the sociodemographic and oncological factors on the decision‐making, we next detected the correlations between variables followed by SEM establishment. All the variables significantly correlated with the independent predictors (Table 2; *P* < 0.05) according to the multivariate regression analysis were selected for the following investigation. The history of abdominal surgery, remarkably correlated with the marital status, was excluded because of the nonsignificance of its association (*P* > 0.05) with the decision on type of reconstruction in the univariate statistics.

**Table 2:** Correlations between study variables (n = 905)

	1	2	3	4	5	6	7	8	9	10	11
1. Age	1										
2. BMI	0.225**	1									
3. Marital status	0.384**	0.185**	1								
4. Residency (economic development)	0.005	0.033	0.062	1							
5. Residency (location)	−0.057	0.050	0.045	0.682**	1						
6. Family history of breast or ovarian cancer	−0.001	−0.038	−0.010	0.035	0.070*	1					
7. History of abdominal surgery	−0.039	−0.016	−0.150**	−0.031	0.006	0.111**	1				
8. Clinical tumour staging	−0.061	0.040	−0.075	0.018	0.029	0.021	−0.032	1			
9. Mastectomy type	−0.003	−0.006	0.013	0.051	0.010	−0.006	−0.028	0.100*	1		
10. Preoperative pathological diagnosis	0.022	0.013	−0.013	−0.032	−0.064	−0.047	0.043	0.122**	−0.089**	1	
11. Neoadjuvant chemotherapy	−0.071*	0.023	−0.021	−0.025	−0.049	0.014	−0.029	0.150**	−0.054	0.048	1

* Correlation is significant at the 0.05 level (two‐tailed).

** Correlation is significant at the 0.01 level (two‐tailed).

**Figure 4:**
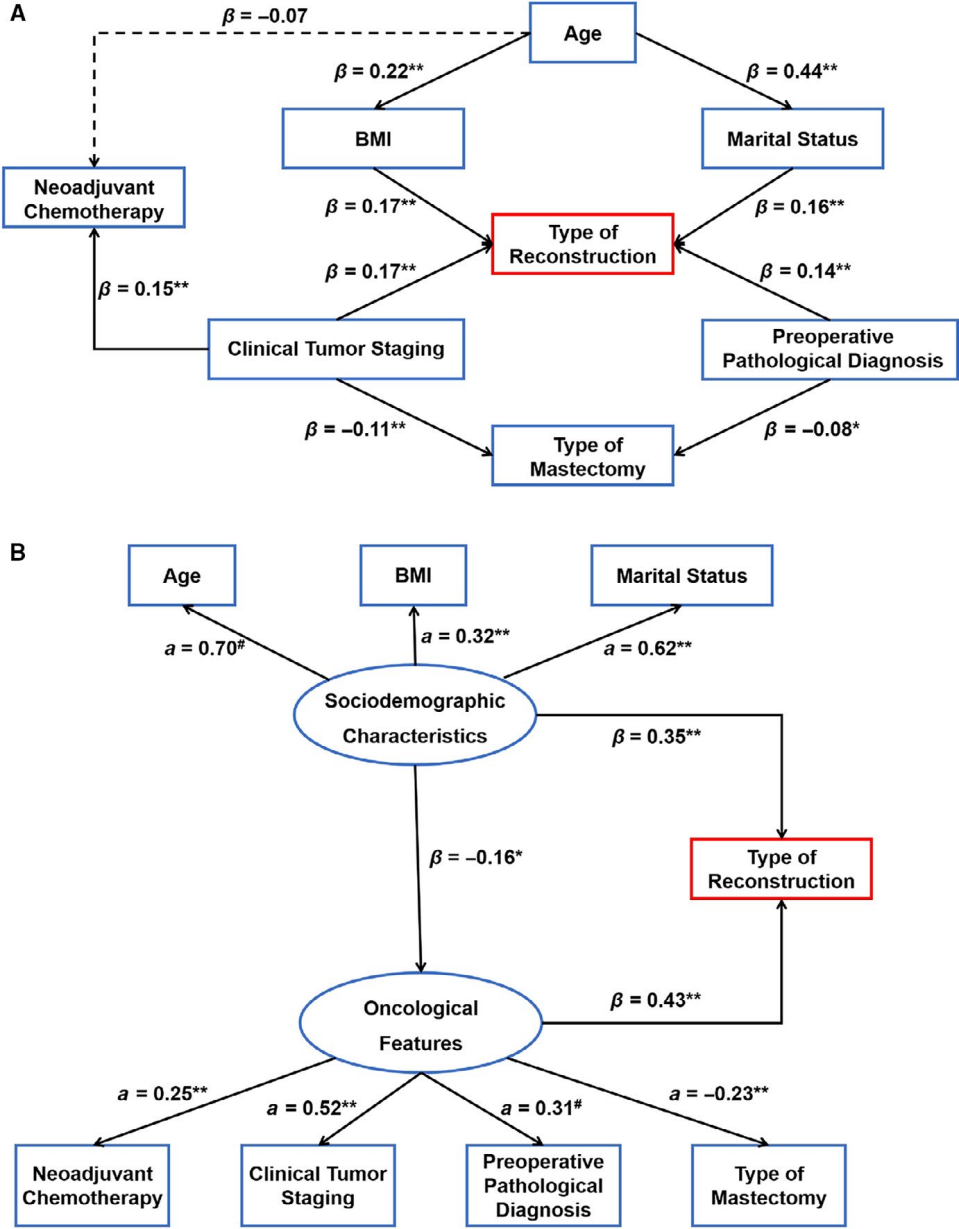
Structural equation models. (A) Hypothesized model. The hypotheses were rejected due to poor model fit. (B) Final model. The model with good fit coefficients shows that the sociodemographic characteristics can represent age, BMI, and marital status; while the oncological features can serve as a proxy for clinical tumor staging, preoperative pathological diagnosis, type of mastectomy, and neoadjuvant chemotherapy. The decision on type of immediate postmastectomy breast reconstruction (IPBR) is significantly affected by the sociodemographic characteristics and oncological features and the impact of the sociodemographic characteristics on the decision‐making is partly mediated by the oncological features. *Note:* β values refer to standardized direct effects on the downstream variables, and *a* values refer to the standardized factor load capacity. The solid lines with single arrow represent significant parameter estimates, and the dotted lines represent nonsignificant parameter estimates. Error variances and covariances are not shown. ^#^factor load was defined as 1.00 in the unstandardized estimates. *P < 0.05, **P < 0.01

The first model (Figure 4A) assumed based on the results of multivariate regression modeling as follows: BMI and marital status may be mediators of the indirect effect of age; patients with diagnosis of noninvasive lesion and earlier clinical tumor stage may be more likely to seek for nipple‐ or skin‐sparing mastectomy; younger age and later clinical tumor stage may be associated with receipt of neoadjuvant chemotherapy. However, it was rejected due to a relatively poor fit with the study data determined as follows: *χ* ^2^ = 45.552, *χ* ^2^/*df* = 2.53, *P* < 0.001; RMSEA = 0.041 (90% CI: 0.026‐0.056); CFI = 0.921. Thus a theoretically and statistically more sound model (Figure 4B) was developed using a combination of confirmatory factor analysis and path analysis, which displayed considerably improved fitting summary: *χ* ^2^ = 25.092, *χ* ^2^/*df* = 1.39, *P* = 0.122; RMSEA = 0.021 (90% CI: 0.000‐0.039); CFI = 0.980. The model suggested that the latent variable of sociodemographic characteristics can represent the observable variables of age (factor load, *a* = 0.70), BMI (*a* = 0.32) and marital status (*a* = 0.62); while the oncological features can be well reflected by clinical tumor staging (*a* = 0.52), preoperative pathological diagnosis (*a* = 0.31), type of mastectomy (*a* = −0.23), and neoadjuvant chemotherapy (*a* = 0.25). Both sociodemographic characteristics (path coefficient, *β* = 0.35) and oncological features (*β* = 0.43) were significantly associated with the decision on type of IPBR (*P* < 0.01). Additionally, the relationship between the sociodemographic characteristics and the decision‐making was proved to be notably mediated by the oncological features (*β* = −0.16; bias‐corrected bootstrap 95% CI of the indirect effect over 5000 iterations, [−0.022, −0.001]; *P* = 0.042). Besides, the moderator effect of the year of surgery on the final model was revealed with no statistical significance (*χ* ^2^ = 7.298, *χ* ^2^/*df* = 2.43, *P* = 0.063).

## 4 DISCUSSION

The clinical practice is fundamentally a decision‐making process based on sophisticated and individualized circumstances.^22^ As for women undergoing mastectomy, IPBR is becoming a popular choice to minimize physical deformity, relieve psychological stress, and optimize quality of life.^14^, ^16^ However, most patients still have to face with a challenging decision on the type of IPBR. Previous investigations have largely focused on the postoperative outcomes of different reconstructive options,^11^, ^12^, ^13^, ^14^, ^15^, ^16^, ^17^, ^23^ which indeed provide empirical evidences for new patients to make a preferred decision. Unfortunately, the association between the preoperative factors and the decision on reconstructive modality remains to be determined, especially for Asian patients. Our present study proves that the decision is not merely a preference‐sensitive one, but a result of multifaceted trade‐offs among the sociodemographic and oncological features before surgery.

A broad array of studies have observed a notable IPBR paradigm shift away from autologous to prosthetic techniques in the United States and United Kingdom over the past decade.^24^, ^25^ A similar trend in China has also been revealed in the current study with an exponentially increasing implant/autologous ratio. The prevalence of implant‐based IPBR in Western countries is reported to be driven by growing use of prophylactic mastectomy,^25^ which may not be the main reason for our findings because the novel technique is still in ethical controversy in China. The sociodemographic characteristics and oncological features are main references for the decision among Chinese patients on the type of IPBR preoperatively, but they are often considered as confounding factors and adjusted for in most investigations. Therefore, we attempted to devise a predicting system by integrating these factors.

The current cohort included all IPBR cases in our institution during the study period. Patients undergoing delayed breast reconstruction were excluded to avoid selection bias for the reason that autologous procedures are more likely to be applied in those cases to resurface the skin defect.^3^ The decision aid process was conducted by well‐trained residents and nurses who complied with the same guideline in order to avoid information bias. The types of IPBR were dichotomized and the delayed‐immediate paradigm was not analyzed as a separate group owing to the limited number of cases. The unpopularity of the two‐stage modality may be attributed to the relatively higher complication rate in Chinese patients who are more prone to have dense parenchyma extending into the adjacent adipose tissue,^28^ which must be radically dissected during mastectomy, leaving extremely thin flaps at the recipient cite. The postmastectomy radiotherapy was not on the list of candidate predictors, although it may induce patients to avoid implant‐based procedures.^5^, ^6^ One explanation is that the receipt of radiation relies on the postoperative pathology of the tumor and axillary lymph nodes which cannot be determined before surgery. Moreover, the preoperative plan for radiotherapy is made based on the clinical tumor staging and preoperative pathological diagnosis, which have already been included in the present study.

The results of univariate analysis indicate that unmarried patients younger than 40 years with BMI ≤ 24 kg/m^2^, early‐stage noninvasive lesions, and bilateral nipple/skin‐sparing mastectomies and without neoadjuvant chemotherapy are more likely to opt for implant‐based than autologous IPBR. Surprisingly, the economic status and residency display little influence on the decision, despite they significantly affect the receipt of breast reconstruction.^24^, ^29^, ^30^ The family history of breast or ovarian cancer, an independent predictor of contralateral prophylactic mastectomy,^31^ also demonstrates nonsignificant association with the decision on type of IPBR. Consistent with previous studies,^32^ the history of abdominal surgery is not a risk factor of autologous IPBR in the current cohort. Moreover, the year of surgery shows remarkable association with the decision, which may be a confounding factor in the present single‐institutional study.

The multivariate regression modeling with adjustment for the year of surgery shows that BMI, marital status, clinical tumor staging, and preoperative pathological diagnosis are independently associated with the decision on type of IPBR. Age does not present direct effect on the decision, which is not in line with the result of a national cross‐sectional study demonstrating younger patients tend to have implant‐based breast reconstruction while older ones are inclined to receive autologous procedures.^33^ The direct associations of mastectomy type and neoadjuvant chemotherapy with the reconstructive procedure choice also approach nonsignificance. However, the indirect effects of those variables are confirmed by SEM. The final model implies that the sociodemographic characteristics and oncological features exert a synergistic effect on the decision on type of IPBR. Furthermore, the oncological features mediate in part the association between the sociodemographic characteristics and the decision. Therefore, holistic evaluation of the preoperative factors, especially the sociodemographic and oncological features, is particularly important to make a high‐quality decision on the type of breast reconstruction.

The current study represents one of the most robust systematic assessments of the association between preoperative factors and decisions on IPBR modality among Asian patients in the available literature, and facilitates understanding the dilemmas in the decision‐making process for both patients and physicians. But this study still has several limitations. First, it is an institution‐based cohort, which may not be generalizable to all patients in all settings, though the cohort is comprised of patients from 29 of 34 province‐level regions in China. Additionally, some factors such as education level, breast size, status of sentinel lymph nodes, etc that may impact the decision on type of IPBR were not included because of massive data missing in the database. Thus future prospective studies are necessary to comprehensively clarify the causal relationship among these factors. The third critique is the observational category of the current study. A randomized clinical trial will be instrumental to design a choice architecture supporting the decision‐making.^34^ Finally, the current study was not designed as an outcome study, although the quality of decision is also an important reference for the patients and surgeons.

## 5 CONCLUSIONS

Among patients undergoing IPBR between 2001 and 2017, the use of implant‐based modality increased exponentially compared with autologous procedures in China. BMI, marital status, clinical tumor staging, and preoperative pathological diagnosis are directly associated with the decision on type of IPBR, while age, mastectomy type, and neoadjuvant chemotherapy are indirectly associated with the decision. The results of the current study add to the limited literature concerning the association of sociodemographic and oncological features with the decision on prosthesis‐ vs autologous‐tissue based IPBR.

## DISCLOSURE

The authors have no financial interest to disclose in relation to the content of this article.
