# Supplementary material for: Association of sociodemographic and oncological features with decision on implant‐based versus autologous immediate postmastectomy breast reconstruction in Chinese patients
Source: Cancer Med. 2019 Apr 5;8(5):2223–32. doi: 10.1002/cam4.2133 (PMC6536967; doi:10.1002/cam4.2133)
Supplement: Supplementary file 1 [file CAM4-8-2223-s001.doc]

**Table S1.** Classification of reconstructive modalities based on *ICD-9-CM-3* codes.

| **Type of Reconstruction** | ***ICD-9-CM-3* code** | **Procedure** |
| --- | --- | --- |
| Autologous Reconstruction | 85.70001 | Total autologous reconstruction of breast |
|  | 85.71001 | Latissimus dorsi flap |
|  | 85.72001 | TRAM flap, pedicled |
|  | 85.73001 | TRAM flap, free |
|  | 85.74001 | DIEP flap |
|  | 85.75001 | SIEA flap |
|  | 85.76001 | GAP flap |
|  | 85.79001 | Other total reconstruction of breast |
|  | 85.84001 | Pedicle graft flap to breast |
|  | 85.85001 | Muscle graft to breast |
| Implant Reconstruction | 85.33001 | Unilateral subcutaneous mammectomy with synchronous implant |
|  | 85.35001 | Bilateral subcutaneous mammectomy with synchronous implant |

***Abbreviations***: TRAM, transverse rectus abdominis myocutaneous; DIEP, deep inferior epigastric perforator; SIEA, superficial inferior epigastric artery; GAP, gluteal artery perforator.
